# “Doctor” or “darling”? Decoding the communication partner from ECoG of the anterior temporal lobe during non-experimental, real-life social interaction

**DOI:** 10.3389/fnhum.2012.00251

**Published:** 2012-09-05

**Authors:** Johanna Derix, Olga Iljina, Andreas Schulze-Bonhage, Ad Aertsen, Tonio Ball

**Affiliations:** ^1^Epilepsy Center, University Medical Center FreiburgFreiburg, Germany; ^2^Faculty of Biology, University of FreiburgFreiburg, Germany; ^3^Bernstein Center Freiburg, University of FreiburgFreiburg, Germany; ^4^Hermann Paul School of Linguistics, University of FreiburgFreiburg, Germany; ^5^Faculty of Philology, University of FreiburgFreiburg, Germany

**Keywords:** natural behavior, temporal pole, theta, alpha, language, speech, BMI, BCI

## Abstract

Human brain processes underlying real-life social interaction in everyday situations have been difficult to study and have, until now, remained largely unknown. Here, we investigated whether electrocorticography (ECoG) recorded for pre-neurosurgical diagnostics during the daily hospital life of epilepsy patients could provide a way to elucidate the neural correlates of non-experimental social interaction. We identified time periods in which patients were involved in conversations with either their respective life partners (Condition 1; C1) or attending physicians (Condition 2; C2). These two conditions can be expected to differentially involve subfunctions of social interaction which have been associated with activity in the anterior temporal lobe (ATL), including the temporal pole (TP). Therefore, we specifically focused on ECoG recordings from this brain region and investigated spectral power modulations in the alpha (8–12 Hz) and theta (3–5 Hz) frequency ranges, which have been previously assumed to play an important role in the processing of social interaction. We hypothesized that brain activity in this region might be sensitive to differences in the two interaction situations and tested whether these differences can be detected by single-trial decoding. Condition-specific effects in both theta and alpha bands were observed: the left and right TP exclusively showed increased power in C1 compared to C2, whereas more posterior parts of the ATL exhibited similar (C1 > C2) and also contrary (C2 > C1) effects. Single-trial decoding accuracies for classification of these effects were highly above chance. Our findings demonstrate that it is possible to study the neural correlates of human social interaction in non-experimental conditions. Decoding the identity of the communication partner and adjusting the speech output accordingly may be useful in the emerging field of brain-machine interfacing for restoration of expressive speech.

## Introduction

There is a long-standing interest in investigating the neural processing of naturalistic sensory stimuli and natural behavior (Aertsen et al., 1981; Montague et al., 2002; Babiloni et al., 2006). An important motivation behind such studies is previous single-neuron research showing that the neural activity in natural, ecologically more valid conditions has different statistical properties than that of artificial stimuli: sparse coding (Vinje and Gallant, 2000; Felsen and Dan, 2005; Yen et al., 2007; Haider et al., 2010), as well as precise (Dan et al., 1996; Mechler et al., 1998; Yao et al., 2007; Haider et al., 2010) and reliable (Haider et al., 2010; Herikstad et al., 2011) spike timing allow conveying information more efficiently. Neural processing in complex, real-life conditions thus cannot be reduced to a superposition of responses to a small set of simple (artificial) stimuli, but likely relies on more complex, non-linear processes [see Hasson et al. (2010) for a review].

Several previous studies on the processing of naturalistic sensory stimuli used natural sounds to explore auditory processing in animals (Suga, 1978; Smolders et al., 1979; Aertsen et al., 1981). In humans, this kind of experiments were adopted (Nelken, 2004) and extended to human-specific stimuli, such as recordings of natural stories (Fletcher et al., 1995; Brennan et al., 2010; Lerner et al., 2011) and movies (Zacks et al., 2001; Bartels and Zeki, 2004; Mukamel et al., 2005; Golland et al., 2007; Privman et al., 2007).

Another line of studies employed non-experimental settings to elucidate the neural basis of unrestrained hand and arm movements in monkeys (Evarts, 1965; Mavoori et al., 2005; Aflalo and Graziano, 2006; Jackson et al., 2006, 2007) and spontaneous, uninstructed language in humans (Towle et al., 2008). Investigations in experimentally unrestricted conditions allow capturing the complexity and functional diversity of real-life behavior more extensively than by standard laboratory procedures (Gibson, 1950) and may prevent a possible contamination of findings caused by the experimental environment as such (Bartlett, 1995), e.g., by the influence of emotional reaction of subjects to the experimenter (Ray, 2002).

Previous studies have also used conditions approximated to real life to study the densely interwoven perception and production processes underlying social interaction in humans. For instance, social interaction has been studied using fMRI experiments in virtual-reality social encounters between subjects and virtual characters (Wilms et al., 2010; Ethofer et al., 2011; Pfeiffer et al., 2011). Also, techniques have been developed to simultaneously record the brain activities of two or more interacting individuals with the help of EEG (Babiloni et al., 2006), fMRI (Montague et al., 2002), and MEG (Baess et al., 2012). In this way, various kinds of interactive behaviors can be investigated, e.g., in spontaneous communication of subjects while playing games (Montague et al., 2002; Babiloni et al., 2006), imitating others' movements (Dumas et al., 2010), or collectively making music (Lindenberger et al., 2009).

Following this trend towards increasingly naturalistic approaches, it would be highly interesting to study brain activity underlying real-life human social interaction outside experiments. This may enable investigators to not only rule out the unwanted effects induced by experimental settings, but, even more so, to investigate the specific kinds of social interaction situations that cannot, or only with great difficulty, be studied experimentally.

**Figure 1:**
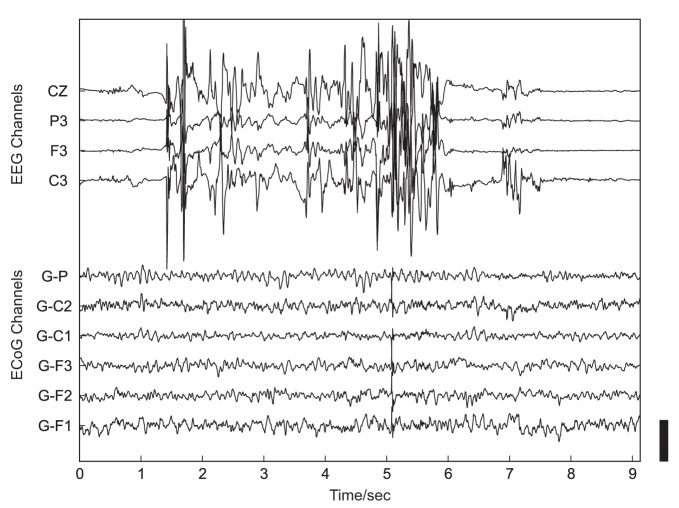
**Example of artifacts related to head movement in simultaneous non-invasive, scalp-recorded EEG (upper 4 traces) and ECoG recorded using subdurally implanted electrodes (lower 6 traces).** The height of the black scale bar in the lower right corner of the plot corresponds to 100 μV.

Such investigations of the neural basis of social interaction in non-experimental, real-life environments are, however, currently lacking (Hari and Kujala, 2009). Major reasons for the absence of such studies are methodological limitations of most recording techniques in humans: traditional imaging methods [e.g., positron emission tomography (PET) or functional magnetic resonance imaging (fMRI)] require a stationary apparatus, with the subjects placed in a fixed position, and therefore these techniques cannot be employed in measurements of dynamic, unrestricted real-life behavior. Non-invasive electroencephalography (EEG) is also not well suited for this purpose due to its limited spatial resolution and its high susceptibility to artifacts, such as those induced by speaking or other movements (Figure 1).

In the present study, we employed, for the first time, human electrocorticography (ECoG) to study neural processes related to real-life social interaction. Owing to the combination of superior temporal resolution and much higher resistance to artifacts compared with non-invasive recordings (see Figure 1 and Ball et al., 2009a), ECoG proved a valuable technique for investigating human motor (Crone et al., 1998a,b) and language (Crone et al., 2001a,b; Sinai et al., 2005) functions, and became a promising candidate signal for clinical brain-machine interface (BMI) applications (Leuthardt et al., 2006; Pistohl et al., 2008, 2012; Ball et al., 2009b), including approaches for restoration of speech production (Blakely et al., 2008; Leuthardt et al., 2011; Pei et al., 2011). In the present study, we performed *post hoc* analyses of ECoG data continuously recorded for pre-neurosurgical diagnostics over several days or weeks during the daily hospital life of epilepsy patients. Throughout the analyzed time periods, patients were conscious, fully alert, and exhibited a wide spectrum of social behaviors, including active interaction with clinical personnel, family, friends, and other patients.

Previous research on social interaction in the fields of linguistics, social psychology, and health care has extensively studied communication between doctors and patients (Roter and Hall, 1989; Ong et al., 1995; Ha and Longnecker, 2010; Nowak, 2011). By contrast, interaction between intimate partners has been within the focus of psychosociological and linguistic research (Sillars and Scott, 1983; Gottman and Notarius, 2000; Pennebaker et al., 2003). Here, we aimed to elucidate, for the first time, the differential neural processes underlying these interactive situations in real-life communication. To do so, we compared conversations during which patients were either talking to their life partners (Condition 1, C1) or to their attending physicians (Condition 2, C2). The two conditions can be assumed to differ in various aspects of social interaction. For instance, patients are more intimate and emotionally attached to their life partners, and share more life experiences with them than with their physicians. Conversely, conversations with physicians are typically more emotionally contained and based on factual communication (Good and Good, 1982).

Our analysis specifically focused on the temporal poles (TP) and the adjacent area of the anterior temporal lobe (ATL) because these areas are associated with several processes crucially involved in social interaction, including autobiographical memory (Spreng et al., 2009), theory of mind (ToM) (Spreng et al., 2009), comprehension of stories (Mar, 2011), and face processing (Olson et al., 2007).

We investigated spectral power modulations in the TP and in the ATL related to social interaction in the alpha (8–12 Hz) and theta (3–5 Hz) ECoG frequency components. Cortical alpha-rhythm changes have been previously associated with dynamic social interaction including eye contact and inter-personal distance (Gale et al., 1975), perception of others' movements (Tognoli et al., 2007), and social coordination (Tognoli et al., 2007; Naeem et al., 2012). Both increases (Gale et al., 1975; Tognoli et al., 2007) and decreases (Boksem et al., 2009) in alpha frequencies have been reported to reflect social cognitive processing. To our knowledge, however, no study has investigated alpha-rhythm modulations in the ATL during social interaction, and it is currently unclear whether alpha power can be employed as a neural marker for social cognition in this brain region. Theta-band changes have been observed in memory-related processes including episodic recollection (Gruber and Müller, 2006), autobiographical memory (Steinvorth et al., 2010), and recognition of familiar faces (Başar et al., 2006, 2007). We therefore expected theta-band power in our target brain regions to undergo modulations by memory-related processing during social interaction.

To estimate the potential usefulness of neural differences during communication with different dialog partners for BMI applications, we also performed a single-trial classification analysis. BMI-based restoration of expressive speech is a topic of growing interest (Pei et al., 2012). So far, BMI studies mainly aimed at decoding such communication-relevant aspects as phonemes (Blakely et al., 2008; Guenther et al., 2009; Brumberg et al., 2011; Pei et al., 2011), words (Kellis et al., 2010), and semantic entities (Wang et al., 2011). Complementary to these approaches, our study makes a first step toward decoding of such high-level information as the identity of the speaker which may help accurate shaping of the language output.

## Materials and methods

### Subjects

**Figure 2:**
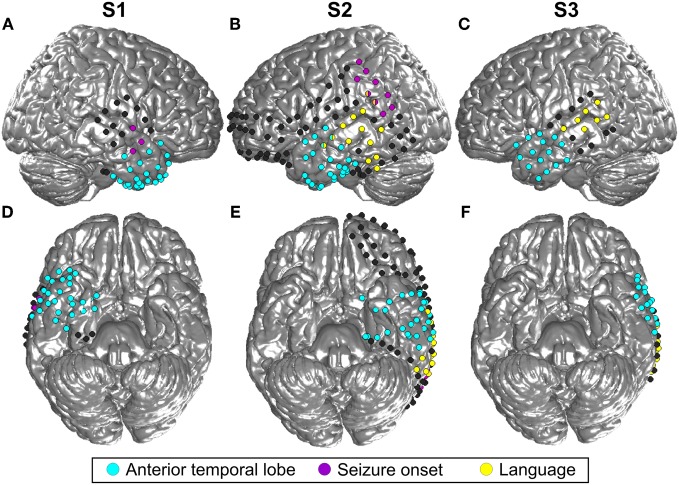
**Location of all implanted grid and stripe electrodes in the three included subjects (S1–3). (A)** is the lateral view of the right hemisphere of S1 and **(B)** and **(C)** show the left hemisphere of S2 and S3, respectively. **(D,E,F)** Display the corresponding bottom sides of the brain. Blue color indicates all contacts located in the ATL, contacts in yellow revealed language functions according to the results of electrostimulation, and contacts in magenta were located in the seizure onset zone. MNI coordinates of the electrodes are projected to an SPM standard brain. For this reason, some contacts which are actually located in the ATL, as indicated by the blue color, may look as if they were situated in the frontal lobe.

Three patients in pre-neurosurgical diagnostics of medically-intractable epilepsy using ECoG were included in this study upon their written informed consent. The study was approved by the Ethics Committee of the University Medical Center Freiburg. Two patients (S1, S3) were right-handed and one (S2) was ambidextrous, all had normal hearing and no history of affective disorders (for more details, see Table 1). Electrode sites analyzed in the present study were outside the seizure onset zone as determined by medical diagnostics. Cortical seizure onset zones in S1 and S2 were in the right posterior superior temporal gyrus and in left parietal areas, respectively, as depicted in Figure 2. In S3, the seizure onset zone was in the left hippocampus and was therefore not visible on the cortical surface.

**Table 1:** **Patient details**.

	**Age (years)**	**Sex**	**Hand.**	**Dom. lang. hem.**	**Seizure onset zone**	**Age epi. onset (years)**
S1	28	f	right	left	right posterior superior temporal gyrus	9
S2	57	m	both	left	left parietal lobe	7
S3	51	f	right	mostly left	left temporal lobe	13

Hand. = handedness, Dom. lang. hem. = dominant language hemisphere, Age epi. onset = age of epilepsy onset.

### Neural recordings

All subjects had subdurally implanted platinum or stainless-steel electrodes (Ad-Tech, Racine, Wisconsin, USA) 4 mm in diameter, covered in sheets of silicone and arranged in regular grids and stripes with a 10-mm center-to-center inter-electrode distance. ECoG was recorded using a clinical EEG-System (ITMed, Germany) at a sampling rate of 1024 Hz, a high-pass filter with a cutoff frequency of 0.032 Hz, and a low-pass anti-aliasing filter at 379 Hz. Digital video recordings (25 Hz frame rate) synchronized to ECoG were acquired for all subjects.

### Conversation periods

Based on ongoing digital video recordings, we identified time periods in which the patients were involved in conversations with their respective life partners (Condition 1; C1) or their attending physicians (Condition 2; C2), see Table 2. The selected epochs contained recordings from time periods during which the patients were having a natural, uninstructed conversation. For all subjects, the length of time periods of speech perception and speech production were roughly balanced between C1 and C2. The position of the conversation partners in the room was not restricted by prior instruction. The patients were sitting or lying in bed with wired connections of electrodes to non-portable amplifiers. During the selected conversation periods, patients were neither eating nor extensively moving their body. The epochs selected by this procedure thus do not necessarily correspond to entire conversations. In the course of conversations, all patients were fully alert, conscious, and able to talk, move, and gesticulate.

**Table 2:** **Electrode implantation and details of analysis**.

	**Impl. hem.**	**No. of ele. in ATL**	**No. of epochs C1**	**No. of epochs C2**	**Total duration of C1 [s] (mean ± std)**	**Total duration of C2 [s] (mean ± std)**
S1	right	27	2	4	266 (133 ± 52.3)	193 (48.3 ± 40.5)
S2	left	30	27	6	6254 (231.6 ± 225.1)	381 (63.5 ± 38.3)
S3	left	15	7	3	883 (126.1 ± 137.5)	149 (49.7 ± 33.8)

Impl. hem., implantation hemisphere; ele., electrodes; std, standard deviation.

### Preprocessing of neural data

For each individual subject, ECoG recordings from all channels were re-referenced to a common average reference of all implanted ECoG electrodes that were located outside the seizure onset zone. For the calculation of time-resolved power spectra, we applied a short-time Fourier transform using successive, non-overlapping, 1-s windows of the recorded ECoG signals, moved in steps of 1 s, resulting in a frequency resolution of 1 Hz.

**Figure 3:**
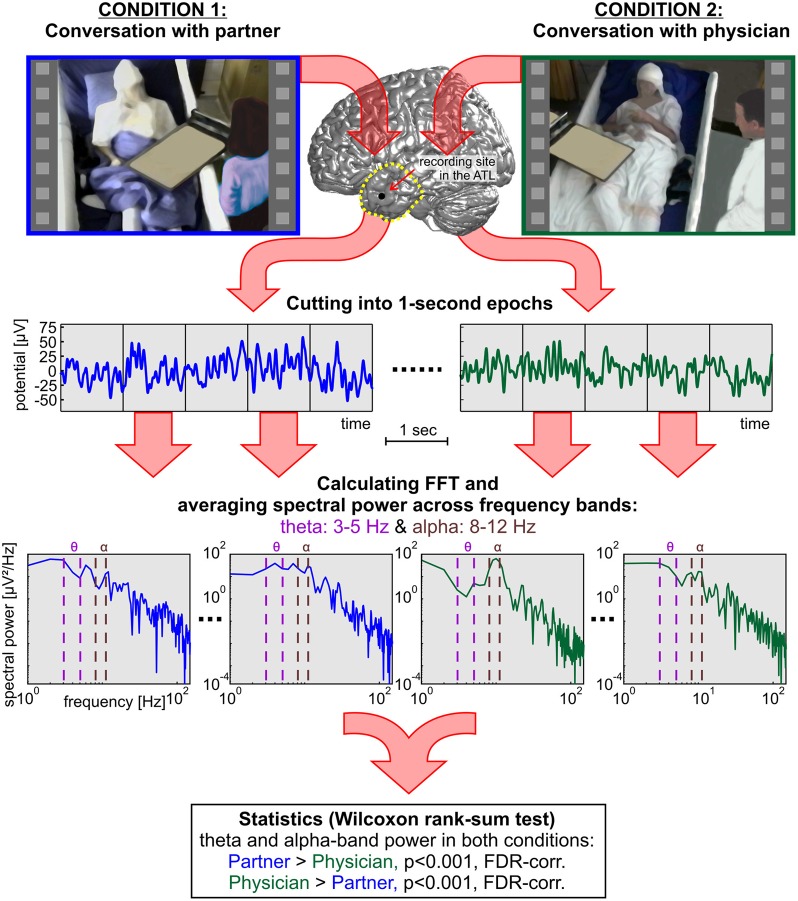
**A systematic overview of the methods applied in the present study to compare neural responses in the TP and in the ATL of three subjects during social interaction with two different dialog partners.** In addition to the rank-sum statistics, single-trial decoding analyses were carried out based on the 1-s epochs.

The hypotheses of the present study refer to modulations in the theta and alpha bands. Therefore, we focused our analyses on these particular frequency ranges. Theta and alpha were defined as the range of 3–5 Hz and 8–12 Hz, respectively. We additionally analyzed the high gamma band in 70–150 Hz, as high gamma is a frequency range that has been extensively studied in previous ECoG research (Crone et al., 1998b, 2001a; Schalk et al., 2007; Ball et al., 2009b). For every channel, the median spectral power for the theta, alpha, and high gamma bands was calculated for each 1-s constituent of the C1 and C2 epochs. For statistical comparison, all power values in the C1 partner condition were tested against power values in the C2 physician condition using the non-parametric Wilcoxon rank-sum test, suited for unequal sample sizes (Sheskin, 2007). Cutting down the sample size of a larger group would decrease the statistical power and is thus not advisable (Rosner and Glynn, 2009). We corrected the resulting *p*-values for multiple comparisons over the number of conditions, channels, and frequency bands (theta, alpha, and gamma) using the false-discovery-rate (FDR) method (Benjamini and Yekutieli, 2001) with a threshold of *p* < 0.001. Figure 3 shows an overview of the computational procedures employed in the present study.

We found that 11 electrodes in S1 showed broad-banded spectral differences across the entire frequency range from 0 to 150 Hz in the two conditions. These channels were not included in further steps of analysis, since such broad-banded responses might be induced by artifacts (e.g., from myographic activity due to head movements) which generally show a broadly and homogeneously distributed frequency spectrum (Kovach et al., 2011). Alternatively, the observed broad-banded changes may arise from unspecific changes of the neural firing rates (Bédard et al., 2006; Miller et al., 2009), representing a different type of response compared to the more narrow-banded spectral power differences investigated in the present study. Such narrow-banded effects (e.g., in the theta or the alpha band) may result from oscillatory mechanisms originating from synchronized neural network activity and may support different dimensions of neural integration, the functional significance of the particular oscillations depending on the brain system involved (Buzsáki and Draguhn, 2004).

To quantify the effect size of the spectral differences between C1 and C2, we calculated in all ATL-electrodes the area under the receiver operating characteristic curve (AUROC) for the theta and alpha-bands separately, using the MES toolbox by Hentschke and Stüttgen (2011).

Single-trial decoding analyses were conducted using a regularized linear discriminant analysis as described in Pistohl et al. (2012). Decoding was performed in each subject separately, based on median (1) theta, (2) alpha, and (3) theta and alpha power values from all available 1-s epochs of C1 and C2. Since theta and alpha signal components may carry complementary information, we used theta and alpha features together in (3). Decoding accuracies were obtained for decoding from all electrodes in the ATL together, as well as for all individual ATL electrodes separately. For the individual contacts, resulting *p*-values were Bonferroni-corrected for multiple testing across the number of analyzed electrodes.

### Electrode positions

Post-operative T1-weighed MPRAGE data sets were acquired for every subject at a 1-mm isotropic resolution using a 1.5-T Vision MRI scanner (Siemens, Erlangen, Germany). The MR images were normalized to a standard brain in MNI (Montreal Neurological Institute) space using SPM5 (Friston et al., 1994). Electrode void artifacts visible in the MR images were identified and marked manually using Matlab programs developed in our laboratory for MRI visualization. Then, the corresponding MNI coordinates of electrode positions were extracted, and individual 3D locations of the contacts were visualized on a standard brain surface. ATL recording sites used for analyses were selected based on the spatial extension of the ATL as illustrated in Figure 3. The TP was defined according to Brodmann's description of area 38 (Brodmann, 1909) as done in Olson et al. (2007).

## Results

C1 and C2 conversation periods were selected according to the criteria described in the “Materials and Methods” section. In subjects S1, S2, and S3, 2, 27, and 7 epochs of conversations with the life partner were available in our monitoring videos with a total duration of 4.4, 104.2, and 14.7 min, respectively. Conversations with the physician could be observed in 4, 6, and 3 epochs for S1, S2, and S3 with a total duration of 3.2, 6.4, and 2.5 min, respectively.

The dialog periods contained intermittent speaking and passive listening, overlapping and non-overlapping talk with different prosodic features of natural discourse, and multiple other aspects of natural oral communication, including conversation fillers, pauses, mimics, and gestures. C1 conversations with life partners covered various topics such as health state, family situation, gossip, news, public events, as well as general reflections about the self and life. In C2 conversations with attending physicians during daily medical rounds, common subjects of discussion were mainly the clinical situation, bodily complaints, progress of the diagnostic process, and small talk, for instance, about an ongoing soccer game and a book. Patients employed the German formal address pronoun “Sie” while talking to the attending physicians, while using the informal “Du” to address their life partners. During the conversation epochs analyzed, the spatial distance between the patients and their dialog partners was on average increased in the C2 condition as opposed to C1.

Of the 61 electrode sites in the ATL included in the whole analyses, 45 electrodes from 2 patients (30 in S2 and 15 in S3) were located in the left, and 16 from S1 in the right ATL (see Figure 2 and Table 2). In total, 25 electrodes were located in the TP, and the majority of all other electrodes were in the temporo-basal part of the ATL. The second most frequent topographical location was the superior temporal gyrus, followed by the inferior temporal gyrus and the middle temporal gyrus (see Table 3 and Table A1).

**Table 3:** **Statistically significant effects (*p* < 0.001, FDR) in the theta (θ), alpha (α), and gamma (γ) frequency bands, MNI coordinates, and anatomical locations of ATL electrodes in S2**.

**Electrode name**	**MNI**			**C1 > C2**	**C2 > C1**	**Anatomical assignment**
	***x***	***y***	***z***			
G_A1	−51	−17	−46	θ, α	−	Temporo-basal
G_A2	−56	−13	−42	−	γ	Temporo-basal
G_A3	−59	−9	−38	α	θ, γ	Inferior temporal gyrus
G_A4	−59	−2	−30	θ, α	γ	Middle temporal gyrus
G_A5	−59	4	−20	α	γ	Superior temporal gyrus
G_A6	−58	10	−13	α	θ	Superior temporal gyrus
G_B1	−55	−26	−44	θ, α, γ	−	Temporo-basal
G_B2	−62	−23	−39	α	γ	Temporo-basal
G_B3	−63	−16	−32	α	−	Inferior temporal gyrus
G_B4	−64	−10	−24	α	γ	Middle temporal gyrus
G_B5	−64	−4	−15	α	γ	Superior temporal gyrus
G_B6	−63	4	−7	α	θ, γ	Superior temporal gyrus
G_C2	−63	−31	−35	α	γ	Temporo-basal
G_C3	65	−24	−24	−	γ	Inferior temporal gyrus
G_C4	−66	−18	−17	α	γ	Middle temporal gyrus
G_C5	−67	−11	−8	θ, α	γ	Superior temporal gyrus
G_C6	−65	−5	−1	α	γ	Superior temporal gyrus
TLA1	−41	7	−55	θ, α	γ	Temporal pole
TLA2	−47	9	−46	θ, α	γ	Temporal pole
TLA3	−50	9	−36	θ, α	γ	Temporal pole
TLA4	−56	10	−25	θ, α	γ	Temporal pole
TBA1	−19	8	−37	θ, α	−	Temporal pole
TBA2	−22	2	−46	θ, α	γ	Temporal pole
TBA3	−28	−2	−53	θ, α	γ	Temporal pole
TBA4	−36	−4	−57	θ, α	γ	Temporal pole
TBB1	−17	−30	−20	θ	γ	Temporo-basal
TBB2	−22	−25	−29	−	γ	Temporo-basal
TBB3	−28	−22	−36	−	θ, γ	Temporo-basal
TBB4	−36	−18	−39	θ	−	Temporo-basal
TBC6	−54	−26	−41	α	γ	Temporo-basal

Electrode names beginning with “G” depict grid electrodes on the lateral ATL, “TL” stands for stripe electrodes in temporo-lateral locations, and “TB” are stripe electrodes in the temporo-basal ATL. For reasons of comprehensiveness, gamma band effects are also provided.

**Figure 4:**
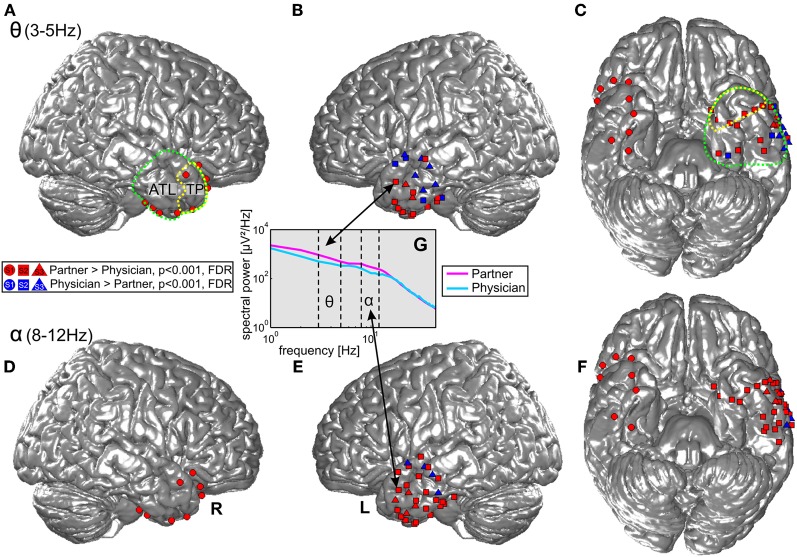
**Projection of ECoG electrode positions on an SPM standard brain. Dots, squares, and triangles depict ECoG electrodes from S1, S2, and S3, respectively.** Red: enhanced activity in the theta (top row) and alpha (bottom row) bands during conversations of the three patients with their life partners (C1) in comparison with conversations with their physicians (C2). Blue: electrodes with significantly enhanced activity in C2 > C1. Green and yellow dotted lines show the anatomical definition of the ATL and the TP. **(A)** and **(B)** display effects in the theta frequency band in the right and left lateral ATL. **(C)** Shows theta effects in the inferior ATL. **(D–F)** Display the corresponding effects for the alpha frequency band. **(G)** Shows an example of one electrode in the ATL with differences in theta and alpha range power in the two conditions.

Statistical tests (*p* < 0.001, FDR-corrected, see “Materials and Methods” and Figure 4) revealed significant differences across the two conditions in both tested frequency bands as shown in Figure 4, Table 3, and Table A1. The spectral power was significantly enhanced in C1 compared to C2 in both theta and alpha frequency ranges in the bilateral TP (15 and 17 electrodes, respectively) and in other parts of the ATL, both on the basal and lateral surface (red markers in Figure 4). In addition, some channels in more posterior parts of the ATL showed reduced activity in C2 compared to C1 (blue markers in Figure 4). Overall, 16 electrodes from the left and 9 electrodes from the right ATL showed significantly stronger spectral responses in C1 than in C2 in the theta range, whereas 28 and 8 electrodes from the left and right ATL, respectively, exhibited enhanced alpha power. For the TP alone, 11 electrodes from the left and 6 from the right hemisphere showed effects in the theta band, and 9 and 6 electrodes from the left and right hemisphere, respectively, showed effects in the alpha band. Conversely, less spectral power in C1 than in C2 could be observed in 10 electrodes of the left ATL for the theta band and in 4 electrodes for the alpha band. In the right hemisphere, there were no electrodes with increased power in C2 compared to C1. All electrodes with less power in C2 than in C1 were located more posterior in the ATL, and none of them was located in the TP. These effects were found in both S2 and S3.

Twenty-six electrodes (22 and 4 for increased C1 and C2, respectively) exhibited effects in the same direction in both theta and alpha bands; 16 electrodes (13 and 3 for increased C1 and C2, respectively) showed isolated effects in the theta or in the alpha band; 3 electrodes revealed reverse theta- and alpha-band changes. From all brain areas with electrode coverage, including large parts of the temporal lobes and parts of the frontal and parietal lobes (see Figure 2 for orientation of electrodes from all subjects), pronounced theta and alpha amplitude differences in C1 compared to C2 were focused on the ATL. The theta and alpha effects in our study were thus both spatially focalized to the ATL (i.e., they did not occur in a spatially diffuse way over all electrodes) and frequency-band-specific.

As mentioned above, although the hypotheses of the present study concerned the alpha and theta bands, we additionally analyzed high gamma activity. In all subjects, most ATL electrodes with significant changes in the high gamma range showed increased power in C2 compared to C1 (43 electrodes), whereas only 5 electrodes exhibited the opposite effect. Electrodes with stronger gamma band power in C1 compared to C2 simultaneously showed significant effects (either increases or decreases) in the lower frequency bands. Thirteen and 23 electrodes with significantly stronger gamma band power in C2 than in C1 at the same time showed decreased activity in theta and alpha ranges, respectively, and increased power was observed in 9 and 4 electrodes in these frequency bands (see Table 3 and Table A1). Yet, effects in the gamma band also occurred in isolation, with no significant differences in the lower frequency bands between the two conditions detectable at the selected significant level.

AUROC values of the ATL electrodes in the theta band ranged across the two conditions between 0.33 and 0.79 in S1, between 0.33 and 0.62 in S2, and between 0.26 and 0.5 in S3. The respective values for the alpha band were between 0.23 and 0.7 in S1, 0.35 and 0.51 in S2, and 0.18 and 0.55 in S3.

We performed single-trial classification of 1-s epochs from C1 vs. C2 for all ATL electrodes together. Decoding from all ATL electrodes based on combined theta and alpha-band power yielded values of 0.67, 0.75, and 0.82 for S1, S2, and S3, respectively. More detailed information, including decoding accuracies for the individual theta and alpha frequency bands, is presented in Table 4. In an analysis based on single electrodes, classification was also above chance significantly (*p* < 0.05, Bonferroni-corrected) in 44% of all ATL electrodes in S1, 46% in S2, and 20% in S3 when decoding was performed based on a combination of theta and alpha frequency bands. Decoding accuracies reached values up to 0.6692, 0.6197, and 0.6788 in S1, S2, and S3, respectively.

**Table 4:** **Results of single-trial decoding of C1 vs. C2 1-s epochs from all ATL electrodes together**.

	**θ**		**α**		**θ and α**	
	**DA**	***p*-value**	**DA**	***p*-value**	**DA**	***p*-value**
**S1**	0.6255	2,67E-03	0.6759	7,35E-09	0.6665	1,35E-07
**S2**	0.7153	0	0.8105	0	0.8414	0
**S3**	0.7297	2,22E-12	0.6770	0	0.7478	0

Corresponding p-values are given based on the theta, alpha, and the combined theta and alpha bands. DA, decoding accuracy.

## Discussion

Human brain processes underlying real-life social interaction in everyday situations have been difficult to study (Hari and Kujala, 2009) and hence remained, until now, a white spot in the literature. In the present study, we moved one step beyond the existing approaches to studying social interaction in near-to-natural experimental conditions by investigating brain activity underlying real-life interaction in ECoG-implanted epilepsy patients under diagnostic monitoring. Epilepsy patients undergoing presurgical diagnostics are in a very specific social situation. Usually, they share rooms with other patients, have a large fluctuation of clinical staff and visitors entering and leaving the room, and are constantly being monitored by video cameras required for this kind of diagnostic procedure. For these reasons, we refrained from calling this situation “natural” and rather employed the term “real-life” to account for the specific circumstances of our patients.

Based on ongoing digital video recordings synchronized to ECoG from 3 patients, we identified time periods in which the patients were involved in conversations with their respective life partners (C1) or with their attending physicians (C2), and compared neural activity in these two conditions as reflected by spectral power in the alpha and theta frequency bands. Both frequency bands showed increased power in C1 compared to C2 in many electrodes located bilaterally in the TP and the entire ATL region. Alpha and theta effects occurred in different combinations, e.g., only in alpha, only in theta, or in both frequency ranges simultaneously. Some contacts in more posterior parts of the left ATL showed opposite effects with significantly increased power in C2 compared to C1. There, modulations of alpha and theta responses sometimes even went in opposite directions at one and the same electrode (Figure 4). These posterior areas might support a different set of cognitive functions which may be recruited more strongly during conversations of the patient with the attending physician. Alternatively, the effects might be linked to inhibitory functional connectivity within an extended cortical network, where increased activity in one node may suppress activity in another, when their coupling is inhibitory.

Conversations between patients and their life partners differ from those with their attending physicians. This becomes apparent from the length and frequency of the interaction periods: indeed, all patients in our study spent much more time communicating with their partners than with the physicians, whom they mostly met during medical rounds for discussing health issues. The TP and the entire ATL region have been associated with the processing of different aspects of social cognition (Olson et al., 2007) and are thus suitable candidate areas for investigating modulations of neural activity related to social interaction. With its widespread connections to other cortical and subcortical areas of the brain (Morán et al., 1987; Kondo et al., 2003), the ATL is a suitable association area for high-level operations to coordinate multiple functions involved in social cognition (Olson et al., 2007). As this part of the brain is topographically remote from primary auditory and visual areas, processing of low-level features is not likely to have affected our results.

An important role in autobiographical memory processing has been attributed to the TP, i.e., recollecting personal events from the life of an individual (Spreng et al., 2009). Autobiographical memories are integral to natural conversation and provide a basis for self-disclosure, entertainment, joint planning and problem solving (Dritschel, 1991). Different social situations involve varying amounts of autobiographical memory, depending on the social distance of the dialog partner and other factors (Dritschel, 1991). Thus, differences in the recruitment of autobiographical memory between C1 and C2 in our study may have played an important role in the strong effects in the TP we observed for C1.

Clearly, social interaction via spoken language also has a linguistic dimension. The ATL region has been associated with language-related processing, including comprehension of narrative speech (Mar, 2011), syntactic complexity of natural stories (Brennan et al., 2010), semantic content (Visser et al., 2010), and narrative context (Xu et al., 2005). As these features may have likely differed between C1 and C2, a possible modulation of the spectral power of the ATL electrodes by such linguistic features is conceivable. A detailed linguistic analysis of the conversation data was, however, beyond the scope of the present study and is a topic for further research.

Another subfunction of social interaction which may have contributed to the observed differential oscillatory modulations in the ATL is the inference of mental states of the dialog partners, a mental function known as ToM. ToM has been associated with processing in the ATL (Spreng et al., 2009; Mar, 2011). Patients can be expected to have more elaborate and consolidated internal models of their life partners than of their physicians that may facilitate the prediction of mental states of the partner (Wolpert et al., 2003). Recognition of various features that are essential to successful interaction may be required to understand another person. An important role has been attributed to the TP and the ATL in recognizing familiar faces (Nakamura et al., 2000; Sugiura et al., 2011), names (Sugiura et al., 2009), and voices (Nakamura et al., 2001) of people. Beyond these specific effects, processing of familiarity in the ATL may be domain-unspecific (Nakamura et al., 2000). Indeed, overarching effects have been shown for personal acquaintances and famous people (Sugiura et al., 2009), familiar faces and scenes (Nakamura et al., 2000), and tools and animals (Whatmough et al., 2002). Evidence for such generality, however, remains contradictory (e.g., Barense et al., 2011).

Since the present study was conducted in epilepsy patients and under non-experimental conditions, it has certain limitations which will be addressed in the following. Although the seizure onset zone in all of our patients was located outside the ATL region (see Figure 2), it cannot be entirely ruled out that our observations may have been influenced by epileptiform activity. Also, we cannot exclude the possibility of epilepsy-related reorganization in our subjects. Therefore, validation of the present findings will be desirable in a sample of epilepsy patients with different seizure origins, as well as in ECoG recordings from subjects with other neural pathologies, such as tumor patients, and confirmation of our results with non-invasive methods in healthy controls will be important.

As electrode placements were defined solely by clinical demands, the three ATL-implanted subjects included into the present study had different electrode coverage. S2 and S3 had electrodes in the left hemisphere and S1 in the right, and, unlike S1 and S2, S3 had no basal electrodes (see Figure 2). These topographic differences have possibly affected our findings. However, differences in the theta and alpha frequencies were consistently observed across conditions and subjects, and could be observed bilaterally both on the basal and lateral surface of the ATL. Since the amount of ATL-implanted subjects available to the present study was limited, we could not systematically address differences across the hemispheres and basal vs. lateral temporal cortex. These interesting topics need to be addressed in future studies based on a larger group of patients.

Another challenge to non-experimental investigation is that we had to rely on the available amount of video-ECoG data. All three patients had longer conversations with their partners than with the attending physicians, who they only talked to during the relatively brief medical rounds. As a consequence, the number of C1-epochs (partner) surpassed that of C2-epochs (attending physician, see Table 2), and this fact had to be considered in the choice of the statistical procedure which had to be suited for group comparisons with unequal sizes (Sheskin, 2007). Furthermore, due to our non-experimental approach, it was not necessarily human interaction alone that may have affected our results. For instance, non-specific effects due to increased arousal/stress levels in the patients while conversing with their physicians might have contributed to the differences of spectral power across the two conditions. In our study, however, the strongest amplitude differences in the lower frequency bands, especially in the alpha range, were clearly focused on the ATL region, which speaks against an explanation of our findings by a spatially global arousal-related modulation of neural activity. A previous study investigating the effect of naturalistic stressors on alpha-range EEG reports modulations in this frequency range to predominate in frontal areas (Lewis et al., 2007) and not in the ATL region, speaking in favor of the view that the spectral power modulations we observed in the ATL cannot be reduced to non-specific arousal-/stress-related effects.

Apart from the regions of interest in the present study, other human brain areas have been associated with the processing of social cognition such as the medial prefrontal cortex, the anterior cingulate cortex, the inferior frontal gyrus, the temporo-parietal junction, and the amygdala (Frith and Frith, 2007). Further studies might reveal novel insights into neural activation in these and other parts of neural networks for social processing with respect to different communicational situations. Investigations may be also extended to other frequency bands. Although the hypotheses of the present study concerned the alpha and theta bands, we additionally analyzed high gamma activity, and typically found increased spectral power in C2 relative to C1. These changes occurred without any strict relation to the changes in lower frequency bands, possibly indicating a different functional contribution of the high gamma band in the investigated brain regions during social interaction. This frequency band has, among other functions, been linked to increased selective attention (Ray et al., 2008), and thus the greater high gamma in C2 might be related to greater attentional demands during conversations with the attending physician. Enhanced power in the high-gamma band in combination with decreased power in the lower frequencies has been previously proposed to indicate increased information processing (Pfurtscheller and Lopes da Silva, 1999). Thus, our observation of stronger power in gamma together with weaker power in the lower frequencies in C2 compared to C1 may also arise from the higher cognitive load during conversations with the attending physician than with the partner.

The two types of social situations involved different degrees of formality: patients addressed their attending physicians in a more official style than they addressed their life partners. Usually, the choice of non-linguistic and linguistic behaviors depend on whom a person is talking to. Such meta-information may be useful for BMI applications aimed at restoration of expressive speech. In natural discourse, decoding whether a BMI user is talking to a stranger, a friend, or an intimate partner may provide helpful information for selecting the style and register to generate the appropriate speech output. Thus, whereas more official, standard language is preferable while speaking to less familiar people and authorities, more colloquial expressions and non-standard language varieties may be favored in conversations with closer people. Context sensitivity may enable the BMI to switch between social situations and select the corresponding mode of speaking. Context sensitivity would thus be a principle to rule out confusion of possibly competitive (e.g., phonetically similar) terms and prevent inaccurate output. For instance, reliable decoding of the C1 and C2 conditions as investigated in the present study from cortical activity in the ATL could prevent a BMI user from startling the beloved person by calling them “doctor” and complimenting the attending physician “darling.”

An interesting step in the present study was hence to investigate whether the identity of the different conversation partners could be decoded from the ECoG signals in the ATL. Based on the theta and alpha frequency components, such decoding was indeed possible in all patients and significance was highly above chance (Table 4). Here, we classified only two communication partners, and future research will be needed to establish whether and to what extent signals from the ATL can be used to extract information about more and other speakers from ongoing activity. Improved classification may be achieved by using alternative brain regions, signal components, and decoding algorithms. Higher spatial resolution using such recording methodology as micro-ECoG (Blakely et al., 2008; Gierthmuehlen et al., 2011; Viventi et al., 2011) will very likely increase the amount of decodable information. We anticipate that decoding of speaker-related information with such optimized techniques may be a valuable contribution to BMI-based restoration of speech in paralyzed patients.

## Outlook

As discussed above, various subfunctions involved in social interaction are likely to have contributed to the observed modulations of neural activity in the present study. Disentangling individual functional aspects that are integral to social interaction will be crucial to address in future research. Many tools are available to characterize different features of real-life behavior at various levels of description. For example, the amount of autobiographical memory units present in natural discourse can be assessed with the system by Dritschel (1991). Many other quantitative systems are available that can be applied to examine human real-life behavior. Thus, the Facial Action Coding System by Ekman and Friesen (1978), available as an automatic tool (Hamm et al., 2011; Maaten and Hendriks, 2011), can be used to infer emotions from facial muscle movements. Approaches from conversation analysis (Sacks et al., 1974), such as the Discussion Coding System, have been utilized to analyze various interpersonal and functional aspects of social interaction (Schermuly et al., 2010). Linguistic methods of discourse analysis can be also applied in neuroscientific research (Brennan et al., 2010), and other aspects of social interaction such as gestures, spatial distance, and body language might be worth investigating. Similar to hyperscanning approaches that employ non-invasive techniques to record simultaneous brain activity from two or more people, even “hyper-ECoG,” or “hyper-ECoG-EEG” studies are conceivable as a way to obtain brain activity measurements from several subjects simultaneously, one or more of them being invasively recorded by means of ECoG.

As discussed in the previous paragraph, a rich spectrum of tools is available that can be applied to refine and extend the real-life ECoG approaches to investigate social interaction. A major purpose of future studies in this direction would be to achieve a better understanding of communication success and failure. Generally, there is much public and scientific interest as to how communicative success during social interaction may affect relationships, e.g., in communication between couples with respect to marital satisfaction (Boland and Follingstad, 1987). Also, several studies showed that specifically for patient-physician interactions, successful communication is causal to patient satisfaction and health status outcome (Stewart, 1984; Jozien, 1991; Staiger et al., 2005). In the present study, we demonstrate that the neural basis of interaction with different communication partners can be traced using ECoG recorded in epilepsy patients. A next step would be to analyze ECoG recordings in epilepsy patients with respect to the success of communication that can be, e.g., quantified using Bales' Interaction Process Analysis (Bales, 1950). This approach might not only reveal the neural signatures of communication, but also provide information that could be used as feedback to improve interaction strategies.

Extraoperative ECoG is a promising candidate signal to study social interaction that may provide new insights into human social cognition. Importantly, such data can be obtained without additional burden to patients and with no need for conducting experiments. A wide range of interaction phenomena and their underlying brain processes can be addressed by means of *post hoc* analyses. This opportunity to investigate brain activity in non-experimental settings may also inspire further experimental studies. Such a combined approach may be particularly helpful to elucidate the neural basis of human social interaction.

### Conflict of interest statement

The authors declare that the research was conducted in the absence of any commercial or financial relationships that could be construed as a potential conflict of interest.

## Appendix

**Table A1:** **Statistically significant effects (*p* < 0.001, FDR) in the theta (θ), alpha (α), and gamma (γ) frequency band, MNI coordinates, and anatomical locations of ATL electrodes in S1 and S3**.

**Electrode name**	**MNI**			**C1 > C2**	**C2 > C1**	**Anatomical assignment**
	***x***	***y***	***z***			
**S1**						
G_A1	39	9	−58	θ, α	−	Temporal pole
G_B1	40	19	−55	θ, α, γ	−	Temporal pole
G_C4	59	13	−21	θ, α, γ	−	Temporal pole
G_D1	33	28	−40	−	−	Temporal pole
G_D2	40	30	−31	θ, α, γ	−	Temporal pole
G_D3	47	27	−23	θ, α	−	Temporal pole
G_D4	56	23	−13	θ, α	−	Temporal pole
TBA1	17	8	−36	−	−	Temporal pole
TBA2	21	7	−48	−	−	Temporal pole
TBA3	29	5	−57	−	−	Temporal pole
TBB1	19	−2	−40	−	γ	Temporal pole
TBB2	27	−5	−49	−	γ	Temporo-basal
TBB3	37	−8	−51	θ	γ	Temporo-basal
TBB4	46	−6	−50	−	γ	Temporo-basal
TBC5	40	−17	−39	θ, α, γ	−	Temporo-basal
TBC6	49	−11	−41	θ, α	−	Temporo-basal
**S3**						
G_A1	−53	23	−14	−	γ	Temporal pole
G_A2	−59	14	−9	−	γ	Superior temporal gyrus
G_A3	−63	4	−4	−	θ, α, γ	Superior temporal gyrus
G_B1	−53	18	−24	−	γ	Temporal pole
G_B2	−58	9	−19	−	γ	Temporal pole
G_B3	−63	1	−13	−	γ	Superior temporal gyrus
G_B4	−66	−9	−7	−	θ, α, γ	Superior temporal gyrus
G_C1	−54	12	−33	α	γ	Temporal pole
G_C2	−59	2	−27	θ, α	γ	Temporal pole
G_C3	−63	−4	−21	−	θ, γ	Middle temporal gyrus
G_C4	−66	−14	−15	−	θ, α, γ	Middle temporal gyrus
G_D1	−55	6	−42	α	γ	Temporal pole
G_D2	−59	−2	−37	θ, α	γ	Inferior temporal gyrus
G_D3	−63	−10	−31	−	θ, γ	Inferior temporal gyrus
G_D4	−66	−19	−25	−	θ, α, γ	Inferior temporal gyrus
